# Invisible or high-risk: Computer-assisted discourse analysis of references to Aboriginal and Torres Strait Islander people(s) and issues in a newspaper corpus about diabetes

**DOI:** 10.1371/journal.pone.0234486

**Published:** 2020-06-11

**Authors:** Monika Bednarek

**Affiliations:** Department of Linguistics and Charles Perkins Centre, The University of Sydney, Sydney, NSW, Australia; University of California Santa Barbara, UNITED STATES

## Abstract

This article employs computer-assisted methods to analyse references to Aboriginal and Torres Strait Islander people(s) and issues in a newspaper corpus about diabetes. The objectives are to identify both the frequency and quality of social representation. The dataset consisted of 694 items from 12 Australian newspapers in a five-year period (2013–2017). The quantitative analysis focused on frequency (raw/normalised) and range (number/percentage of texts). The qualitative analysis focused on the identification of semantic prosody (co-occurrence with negative/positive words and phrases) and on selective social actor analysis. The qualitative analysis also compared choices made by the press to language practices recommended in relevant reporting guidelines. Key results include that references to Aboriginal and Torres Strait Islander people(s) or matters appear to be extremely rare. In addition, newspapers’ language choices only partially align with guidelines. References that do occur can be classified into four categories: a) references to [groups of] people and other references to identity; b) names of services, institutions, professions, roles etc; c) non-human nouns related to health; d) non-human nouns related to culture. Qualitative analysis of the word COMMUNITY suggests that newspapers for the most part do recognise the existence of different communities at a national level. However, analysis of all references to [groups of] people shows that the vast majority occur in contexts to do with negativity, therefore having a negative semantic prosody. More specifically, there is a strong association with mentions of a higher risk, likelihood, or incidence of having or developing diabetes (or complications/effects). In sum, Aboriginal and Torres Strait Islander people(s) and issues lack in visibility in Australian diabetes coverage, and are associated with deficit framing, which can be disempowering. To change the discourse would require both an increased visibility as well as changing the deficit lens.

## Introduction

The use of language matters. It’s important to remember that for many Indigenous people, this is not just a story, a newsgrab, a headline. Indigenous narratives are often complex and nuanced, so journalists should … be mindful of how language is used and contextualised. [1, p. 4]

This piece of advice comes from recently published guidelines, and brings home the message that ‘language matters’. This article heeds this message and focuses on how language is used in Australian national and metropolitan newspapers in a case study on diabetes.

Why diabetes? Diabetes is a major health problem in Australia, including for Aboriginal and Torres Strait Islander people [2–3]. Statistically speaking, diabetes is the second leading cause of death for Aboriginal and Torres Strait Islander people–compared to seventh for all Australians [4]. In fact, diabetes is a major contributor to the 10–20 year difference in life expectancy [5], a difference that is more generally affected by racism and the ongoing effects of colonisation in Australia which impact negatively on health [6–10].

In general, the media influences *what* is considered important and worth reporting (‘agenda setting’; see [11]), as well as *how* an issue is reported (‘framing’; see [12]). In the context of diabetes, the media can influence who is seen as responsible, both in cause and solution [13]. Further, health news coverage can affect public policy agendas, policy makers, and the policy process [14–17], with potential effects for funding and research. The news media have therefore been widely recognised as an influential source of information related to health in general (e.g. [14], p. 159) and to Aboriginal and Torres Strait Islander health in particular (e.g. [16–18]).

So how visible are Aboriginal and Torres Strait Islander people(s) and issues in diabetes coverage, and how is language used in such coverage? In this article, an approach called *corpus linguistics* is adopted to answer these questions. Corpus linguistics–computer-based linguistic analysis–involves researchers using specialised software to analyse datasets both quantitatively and qualitatively. This study focusses on a five-year dataset of almost 700 newspaper articles published between 2013 and 2017. It is, to my knowledge, the first-ever study to use a corpus linguistic approach to analyse media representation of Aboriginal and Torres Strait Islander people(s) and issues. However, corpus linguistics has been widely used to analyse representations and discourses in relation to other topics and news actors in the news, in an approach variously called *corpus-assisted discourse studies* or *corpus-based (critical) discourse analysis* [19].

While I draw on the above-cited reporting guidelines in this article, it is important to note that these guidelines were only published in 2018. Although the effectiveness of these guidelines is therefore not tested (à la [20] in relation to diabetes), the results of this study can provide a baseline for future research that aims to do so. In addition, it is important to emphasise that not everyone will agree on the recommendations in the guidelines. As they state:

Aboriginal and Torres Strait Islander peoples are a diverse group of people so opinions on what is the most appropriate terminology is varied. This may also change overtime, and you will never have clear-cut ‘correct’ terminology to use, the best way to know what is appropriate is to keep asking.

[1, p. 4]

There has been some recent research on language use in diabetes coverage in the Australian print media, but it did not include Aboriginal and Torres Strait Islander people(s) or issues [20]. This research does argue, however, that ‘the language used by the media in communicating about diabetes has the potential to influence general perceptions and attitudes about diabetes in a whole spectrum of people, not just those people living with diabetes but also their spouses, friends, parents, and significant others’ [20, p. 493]. Another study [21], which this article builds on and extends, only reported the low number of references to Aboriginal and Torres Strait Islander people(s) or matters in diabetes coverage alongside other project results (described in more detail below in ‘Frequency and range’). There is a general paucity of research on Australian media representations of Indigenous health [9, 17], especially in relation to language use. However, relevant studies on nutrition [18] and obesity [9] find low coverage in terms of number of articles published and identify topic-specific frames (e.g. causes of obesity or policy functions). For particular newspapers, coverage of Indigenous health is low and only occurs in moments of intense crisis, although both amount and framing vary across newspapers and over time [16–17]. In general, it has been argued that mainstream media coverage of Aboriginal and Torres Strait Islander health is characterised by an overarching crisis or deficit frame–a focus on negativity, failure, crisis, disadvantage, and dysfunction [16–18, 22–24].

## Data and methods

### Data

The dataset analysed in this article is the Diabetes News Corpus (DNC) and consists of items that were published in 12 Australian newspapers in the last five years preceding the start of the project (Table 1). Where available, newspapers were chosen from both of Australia’s main news organisations Fairfax and NewsCorp, and Sunday editions were included. In order to capture a wider and more representative set of data, it was decided to take a state- and territory-based approach rather than focussing just on the top two by circulation, as done in [20]. This means that we are able to consider newspapers published across states and territories in Australia. The focus, however, remains on the major ‘mainstream’ outlets, rather than focussing on Indigenous or local/regional publications. The DNC is available for other researchers through an online platform (http://cqpw-prod.vip.sydney.edu.au/CQPweb/), which allows researchers to just focus on particular publications, so that comparisons with other datasets are possible.

**Table 1:** Newspapers in the dataset.

National	*The Australian Financial Review; The Australian*
New South Wales	*The Sydney Morning Herald* (and *The Sun Herald*); *The Daily Telegraph* (incl. *Sunday Telegraph*)
Victoria	*The Age* (and *The Sunday Age*); *Herald Sun* (incl. *Sunday Herald Sun*)
Australian Capital Territory	*The Canberra Times*
West Australia	*The West Australian*
Northern Territory	*The Northern Territory News* (incl. *Sunday Territorian*)
Queensland	*The Courier Mail* (incl. *Sunday Mail*)
Tasmania	*The Mercury*
South Australia	*The Advertiser*

Detailed information about the design and building of this dataset is provided in [25]. To briefly summarise here, an online database was used to retrieve any items that include diabet* (any word starting with *diabet*, i.e. *diabetes*, *diabetic*, *diabetics*, *diabetic’s*) in the headline or lead paragraph from 1 January 2013 to 31 December 2017. Identical items within the same newspaper were excluded, whereas identical items published in different newspapers were not excluded. Partial duplication (e.g. using the same quotes from the same sources or the same paragraphs from a media release) also occurs across non-identical articles (see also [20], p. 496). Such items are also included.

Certain items were excluded automatically through Factiva’s optional news filter (e.g. obituaries, captions, letters, food items). Each item in the search results was then surveyed manually to exclude further items following Gounder & Ameer’s criteria [13, p. 6]–namely, discarding short articles (fewer than 150 words), articles that discuss diabetes in non-humans, and articles that only mention diabetes in passing (details in [25]). The final dataset comprises 694 articles, including both news and ‘non-news’ items (as operationalized in [25], for example opinion, analysis, profiles). Table 2 shows the word count for each section and the whole corpus. In this article I focus on diabetes coverage as a whole and do not systematically distinguish between the two subsets. There are several reasons for this: i) I am interested in language use in newspapers in general, ii) both news and other text types are part of the media landscape in which relevant policies and decisions are made, iii) the small dataset and relative lack of language about Aboriginal or Torres Strait Islander people(s) and issues means that a restriction to one sub-set would result in much fewer data to analyse.

**Table 2:** The Diabetes News Corpus.

Dataset	Tokens (running words) in text^a^
News sub-set	193, 659
Non-news sub-set	56,315
Whole corpus (DNC)	249,974

^a^ Settings: hyphens separate words; characters within word:’.

In addition to the DNC, a second dataset is used for occasional comparison, called NOW-OZ. NOW-OZ is a subset that was created using data from the NOW corpus [26], a large dataset of texts from online magazines and newspapers in 20 English-speaking countries. For the NOW-OZ corpus, only texts from Australia between 2013 and 2017 were included (353,802,816 tokens). This corpus will inform us whether language use in the DNC is similar to language use in the Australian media more generally. (For copyright reasons, every 200 words ten words of the NOW texts are replaced with ‘@’, which affects all words equally; see https://www.corpusdata.org/limitations.asp).

### Methods

**Fig 1:**
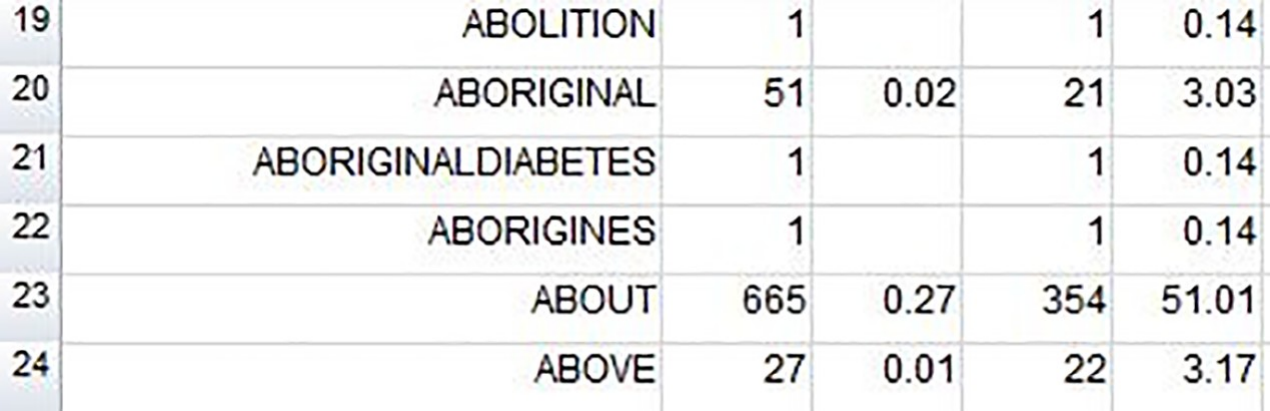
Extract from the alphabetically-sorted frequency list.

Files in the Diabetes News Corpus were cleaned, split into separate items, and turned into plain text files for processing using WordSmith [27]–a specialised computer program which enables identification of word forms and their frequency in and across texts in a dataset (through the WordList function). A word list can be sorted by frequency (the most frequent word form is at the top) or alphabetically (words starting with *a*- are at the top). The latter allows for the search and identification of particular word forms (e.g. *Aboriginal* or *Indigenous*), as illustrated in Fig 1.

The columns next to the word forms in Fig 1 show the raw frequency (e.g. *Aboriginal* occurs 51 times), the frequency as a percentage of the running words in the corpus (e.g. *Aboriginal*: 0.02%), the range or number of texts in which the form occurs (e.g. *Aboriginal* occurs in 21 texts out of a total of 694), and the percentage of texts in which it can be found (e.g. *Aboriginal*: 3.03%).

**Fig 2:**
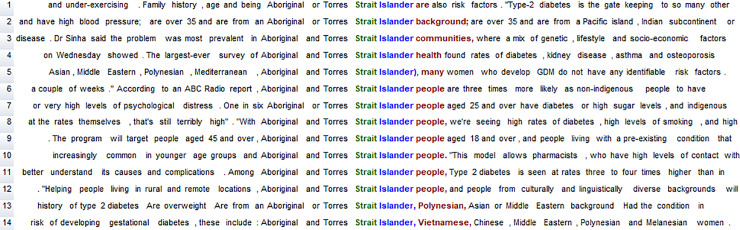
Concordance lines for *Aboriginal and/or Torres Strait Islander* in the DNC.

The WordSmith program also includes a Concordancer (Concord) which allows manual inspection of each word form for qualitative analysis, for example to ascertain how a word such as *Islander* is used (Fig 2). It thus becomes possible to combine quantitative and qualitative analysis. Importantly, concordance lines can be sorted alphabetically (to the right, to the left, or a mix of both), which enables the identification of linguistic patterns. For example, the alphabetic sorting in Fig 2 shows the nouns that are pre-modified by *Aboriginal and/or Torres Strait Islander* (e.g. *background*, *communities*, *health*, *people*) as well as other uses of this phrase.

To interpret results, I loosely draw on the corpus linguistic concept of semantic prosody [28]. The term is used here to refer to *POS/NEG collocation* [29]–the co-occurrence of a word with a set of words or phrases that are positive or negative. When a word or phrase is categorised as positive or negative, it is either attitudinal (expressing speaker opinion) or an instance of positive/negative lexis, i.e. ‘expressions that describe negative [or positive] events or news actors, but that do not explicitly inform the audience that the writer disapproves [or approves] of them’ [30, p. 86]. In this article, close reading of concordance lines is preferenced over the identification of co-occurrence patterns through statistical association measures. Further, the analysis is not restricted to individual word forms in a pre-set span, but rather considers the surrounding text (co-text) more broadly. For example, the sentence *The rate of diabetes in Aboriginal people was three times as high than in non-Indigenous people* would be considered as an instance where *Aboriginal people* occurs in a negative co-text. It must be emphasised that the identification of surrounding text as positive (e.g. *improvements*) or negative (e.g. *death rate*) is based on the researcher’s assessment. For this reason, the full analysis is available in S1 Table.

I also refer to selected categories of van Leeuwen’s social actor network [31], which presents choices that can be used in texts to represent social actors. For example, social actors can be classified (through language) in terms of identity categories such as gender or race; they can be referred to as individuals or as groups; and they can be associated with other social actors or differentiated from them. Relevant choices will be explained below in the discussion of results.

## Results and discussion

### Frequency and range

As a first step, the alphabetically-sorted DNC word list was consulted to identify any potential references to Aboriginal and Torres Strait Islander people(s) and matters–as briefly summarised in [21] and elaborated on here. As a reminder, a word list is based on the occurrence of single forms; that is, it will list *Torres*, *Strait*, and *Islander* separately. Table 3 shows identified word forms, together with their raw frequency, and range. At a basic level, words such as *Aboriginal* and *Indigenous* are in themselves used for *classification* [31, p. 42], identifying social actors on the basis of a major identity category. It can be argued that these words ‘oversimplify the hundreds of nations that exist within Australia’ [1, p. 4], and it is recommended to ‘identify Aboriginal and Torres Strait Islander people in as specific a manner as they are comfortable with—i.e. by people/nation or language group.’ [1, p. 4]. However, a search for *woman*, *man*, *person*, *people*, *language*, *clan*, *band*, *mob*, *nation*, *community*, *communities*, *country*, *land*, *heritage* and *ancestry* did not identify any co-occurring names for specific Aboriginal or Torres Strait Islander language groups, clans, or nations in the DNC. Likewise, a search for *first* did not retrieve any instances of *first nation*(*s*) or *first people*(*s*). (One caveat: it is theoretically possible that texts include Aboriginal and Torres Strait Islander people without identifying them explicitly as such, i.e. not using any identity label. To find out if this is the case, all 694 articles would need to be read and all referenced social actors would then have to be researched as to their ethnicity–a highly time-consuming method which could not be pursued.)

**Table 3:** Word forms in the word frequency list.

Word form	Raw freq.	Range	
		Texts	Percentage
ABORIGINAL	51	21	3.03
ABORIGINALDIABETES	1	1	0.14
ABORIGINES	1	1	0.14
INDIGENOUS	69	23	3.31
TORRES	14	11	1.59
STRAIT	14	11	1.59
ISLANDER	15	12	1.73

Table 3 shows that the raw frequencies are small, the word forms each only occur in 1.6–3% of the data, and are not very well distributed across the corpus. The form *Aboriginaldiabetes* is not a typo or software processing error, but rather occurs in an internet address. Because of the software settings, Table 3 includes one instance of *non-Aboriginal* and 14 instances of *non-Indigenous*. Disregarding these, there are 50 instances of *Aboriginal* and 55 instances of *Indigenous* in the corpus.

The fact that Table 3 includes 14 instances of *Torres* and *Strait* suggests that there are 14 instances of *Torres Strait Islander* (see also Fig 2 above). Concordancing of *Islander* confirms this, also identifying one additional occurrence of *Pacific Islander*. While there is one instance of *Aborigines*, the singular *Aborigine* does not occur (see also Fig 1). Both forms–*Aborigine* and *Aborigines*–are dispreferred, according to the guidelines:

…the terms “Aborigine/s” or “Aboriginals” have negative connotations and are highly offensive to many. Historically, it has been used in racist contexts as a derogatory term to belittle or objectify Indigenous people. For these reasons, it best to avoid using such terminology. [1, p. 5]

In this respect, it is important that the one instance in the DNC is not a historical reference but occurs in an article from *The Advertiser* in November 2015. It is not attributed to a source, but rather originates in the institutional voice of the newspaper:

SAHMRI’s Aboriginal Diabetes Study is now recruiting 4000 **Aborigines** across South Australia to take part, 2000 with the disease and 2000 who do not have it.

The alphabetically-sorted word list also showed that abbreviations of the words *Aboriginal* or *Torres Strait Islander* (e.g. TSI, ATSI) do not occur in the DNC, which is a positive finding:

Aboriginal should never be abbreviated and Torres Strait Islander should be used in full and not shortened to ‘TSI’. In the same vein, don’t shorten Aboriginal and Torres Strait Islander peoples to ATSI, unless of course it’s part of an acronym of an organisation. [1, p. 5]

The guidelines also propose that the expressions *Indigenous*, *Aboriginal* and *Torres Strait Islanders* should start with a capital letter (p. 5). (In this article, initial letters are capitalised, unless text passages/examples from the newspapers are quoted.) Concordancing shows that all instances of *Aboriginal* and *Torres Strait Islander* in the DNC are capitalised in this way. However, the vast majority of instances of *Indigenous* are not dignified with a capital ‘I’: one instance of *non-Indigenous* and 11 instances of *Indigenous* are capitalised (including instances at the beginning of sentences and in all caps), while the remainder are not (57 instances, including 13 occurrences of *non-indigenous* and four instances that refer to Indigenous groups/peoples/diets around the world). The uses of these forms vary, and include adjectival premodification of non-human nouns, such as *indigenous central Australia*, *indigenous diabetes*, or *indigenous health*–a finding to which we will return later. (These results assume that the way the texts are preserved in Factiva is identical to how they appear in the print versions. A spot-test was undertaken, checking the Factiva version of one article from nine different newspapers against their respective hard copy print versions available on micro-film in the State Library of NSW. Of 34 instances of *Indigenous* (including *non-Indigenous*) in these nine articles, 100% matched the capitalisation in Factiva, i.e. the capitalisation in the DNC).

**Fig 3:**
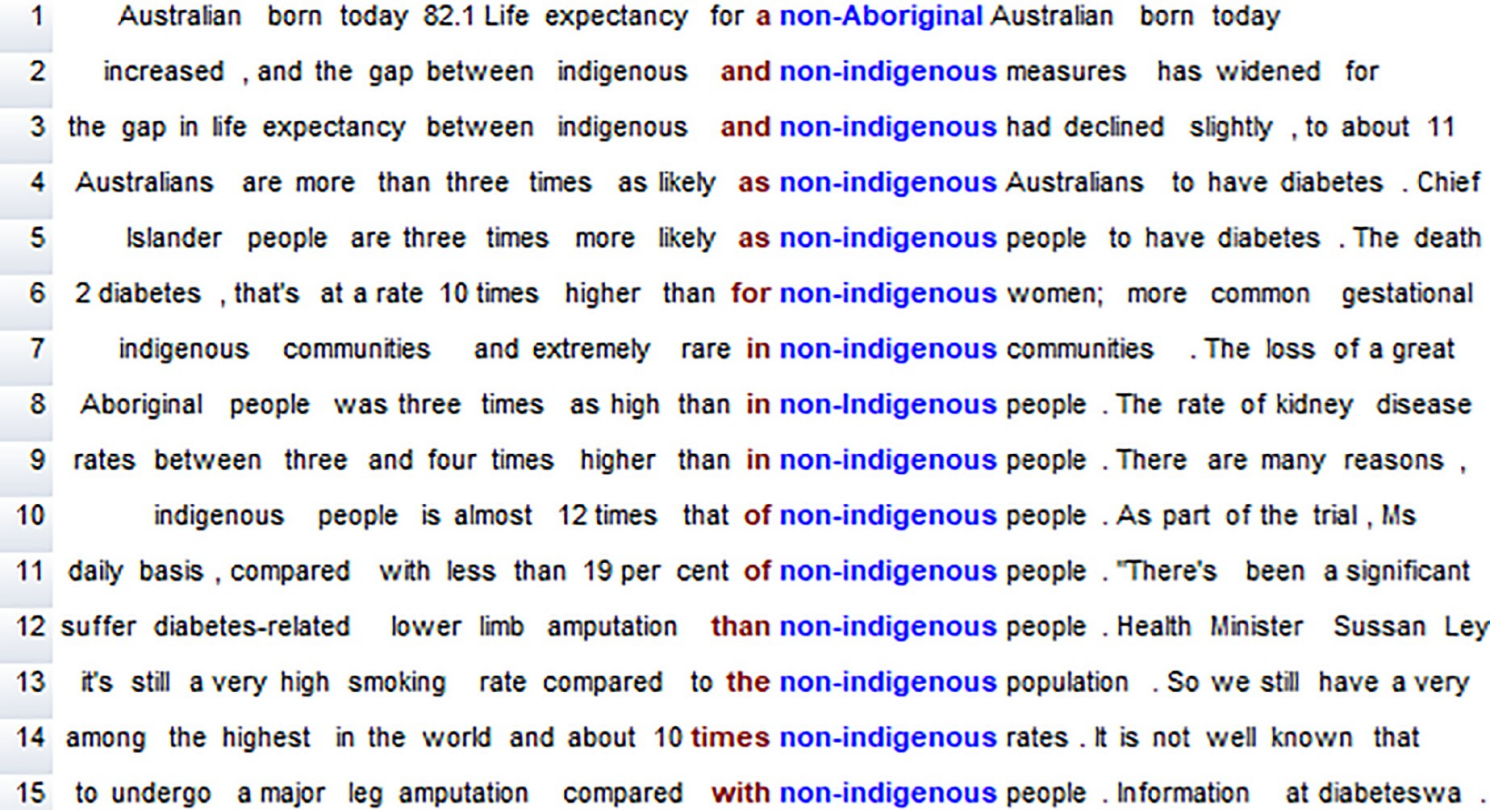
Instances of *non-Aboriginal* and *non-Indigenous* in the DNC.

Since Table 3 lists the range of all word forms separately and also includes instances of *non-Aboriginal* and *non-Indigenous*, the Concordancer was used to first search for any occurrence of any of the forms *Aboriginal*, *Aborigines*, *Indigenous* or *Torres Strait Islander*, and to then exclude any instances of *non-Aboriginal* or *non-Indigenous*. (These specific forms were chosen because of the word frequency results reported above. The search string was ‘Aboriginal/Aborigines/Indigenous/Torres Strait Islander’, where the slash stands for ‘or’ in the relevant search syntax). In fact, the word forms *non-Aboriginal* and *non-Indigenous* occur in the same articles as their counterparts, in comparisons (Fig 3). In other words, an explicit difference is created between two groups that are thus implicitly presented as homogenous (*differentiation* in van Leeuwen’s terms [31, p. 40]). Such differentiation has the potential to reinforce essentialist discourses that represent Aboriginal and Torres Strait Islander Peoples as a single homogenous group, an ‘Other’, different from ‘us’ (identified as a theme in Australian politicians’ statements in [32] and in the media in [22]).

The results indicate that only 36 out of a total of 694 items (about 5%) in the DNC include an instance of *any* of the four search terms. The low coverage is in line with results concerning coverage of obesity [9] and nutrition [18]. Among these 36 articles (26 news; 10 non-news), two pairs of articles are near-identical duplicates, with another two articles (by the same author) containing a considerable overlap in vocabulary. In fact, matters are worse since close reading of these articles shows that only eight are primarily about Aboriginal or Torres Strait Islander people(s) or issues (7 news; 1 non-news). Most of these eight articles do cite at least one Aboriginal or Torres Strait Islander person, with the exception of one news item about a missing person (citing police only) and one non-news item. The remaining 28 articles include three that cite an Aboriginal or Torres Strait Islander person. Cited sources are either people who live with diabetes (e.g. *Alice Springs mother Nellie Impu*), people who speak in a professional capacity (e.g. *Lisa Jackson Pulver*, cited in her role as Professor of Public Health and Director, Muru Marri Indigenous Health Unit, UNSW) or both (e.g. an Aboriginal liaison officer, who is also living with diabetes). To analyse whether the number of Aboriginal voices has increased in the Australian print media since the 1990s [33], further research is necessary specifically into sourcing, to determine the proportion, status (e.g. elite vs ‘ordinary’), style (e.g. direct vs indirect quotation) and content (e.g. personal opinion vs analysis) of cited news sources in relation to a wide range of topics. Some evidence for a positive shift comes from a study on nutrition stories [18, p. 281] and a study on obesity [9, p. 8], both of which identified Indigenous voices (e.g. stakeholders, spokespeople, program coordinators, health workers, academics, celebrities) as common in recent health coverage.

The first significant result of this study, then, is that references to Aboriginal and Torres Strait Islander people(s) or matters appear to be extremely rare in diabetes coverage. This tendency is problematic, because it means an absence or invisibility from public discourse aimed at Australian audiences.

### Uses of *Aboriginal* and *Indigenous*

So far, we have mainly considered the frequency and range of particular word forms and we will now turn to how these word forms are used. The guidelines [1] note that some people disprefer the word *Indigenous*, because of its lack of specificity. They state:

If in doubt, the most common and widely used terminology is “Aboriginal” or “Torres Strait Islander” peoples. “Peoples” is often used instead of people to stress that both Aboriginal and Torres Strait Islander communities are made up of distinct nations, clans and language groups. The National Congress of Australia’s First Peoples advocates for journalists to only use tAboriginal [sic] and/or Torres Strait Islander/s or Peoples when reporting. […] If you are wanting to say “Aboriginal and Torres Strait Islander peoples” more than once in a story, then it is common practice to subsequently refer to “Indigenous people”. [1, p. 4]

Aldrich et al, citing [34], suggest that *Indigenous* is ‘a term of convenience which, by failing to acknowledge diversity, characterises Aboriginal and Torres Strait Islander Peoples as a homogenous “Other”‘ [32, p. 135]. Other guidelines also suggest that *Indigenous* is dispreferred, but can be used ‘where Aboriginal and Torres Strait Islander is used repeatedly within a shorter document’ [35, p. 2]. (This article similarly uses *Indigenous* to avoid too much repetition of *Aboriginal and/or Torres Strait Islander*.)

**Fig 4:**
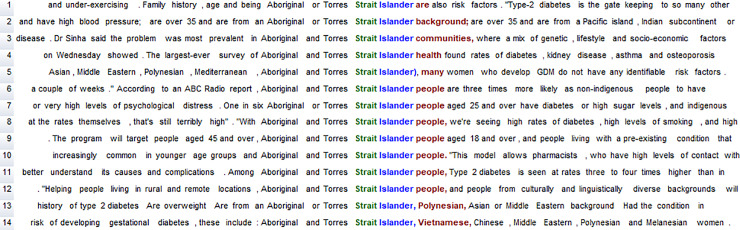
Instances of *Aboriginal and/or Torres Strait Islander*.

Examining first the 50 instances of *Aboriginal* (excluding *non-Aboriginal*) in the DNC, only 14 occur as part of *Aboriginal and/or Torres Strait Islander* (Fig 4). No instances of *Aboriginal* are noun uses, which is a positive finding (c.f. Aboriginal and Torres Strait Islander Guide to terminology, no date: 1).

With respect to the use of the singular *people* vs. the plural *peoples*, public health guidelines [35, p. 2] distinguish between references to individuals (singular) and nations (plural), which seems to be in line with the usage here. There is only one instance of *peoples* in the DNC, and this refers to *indigenous peoples around the world*. However, the expression *Indigenous people* is only sometimes used together with mentions of *Aboriginal* or *Aboriginal and Torres Strait Islander* in the same text. In most cases, this expression is therefore *not* used to avoid too much repetition of *Aboriginal* or *Aboriginal and Torres Strait Islander* and could be rewritten using other referring expressions.

We will now look in more detail at how the words *Indigenous* and *Aboriginal* are used in the DNC (including *Aboriginal and/or Torres Strait Islander*, but excluding *non-Aboriginal* and *non-Indigenous*). (S2 Table lists all identified uses together, categorised according to form and ranked by frequency. For the sake of completeness, even instances that occur only once are listed. Note that Fig 3 above shows that several of the identified nouns also co-occur with *non-Aboriginal* and *non-Indigenous*, namely, *Australian*[*s*], *communities*, *people*, *population*, *measures*, *rates*, *women*). These uses can be classified into four larger categories:

- References to [groups of] people (e.g. *Aboriginal community*; *Aboriginal and Torres Strait Islander people*; *Aboriginal women*; *an indigenous child*) and other references to identity (e.g. *we are indigenous*; *are from an Aboriginal or Torres Strait Islander background*; … *of Aboriginal appearance*)
- Names of services, institutions, professions, roles etc (e.g. *the Aboriginal Medical Services Alliance Northern Territory*; *Wardliparingga Aboriginal Research Unit*; *an Aboriginal liaison officer*; *Aboriginal healthcare workers*; *Aboriginal Health Theme Leader*; *federal Indigenous Health Minister*; *director of Muru Marri indigenous health unit*)
- Non-human nouns related to health (e.g. *Aboriginal and Torres Strait Islander health*; *Indigenous diets*; *Indigenous smoking rates*)
- Non-human nouns related to culture (e.g. *Aboriginal rock art*; *Indigenous language groups*).

I will focus below in more detail on the larger category of references to [groups of] people, but will briefly comment on three specific cases.

First, references to identity that use the word BACKGROUND (the lemma, i.e. including all forms of the word such as singular and plural), tend to be used for what van Leeuwen calls *association* [31, p. 38]–the creation of groups in texts by association with each other (for example through use of *and* or *or*). Examples are provided in S2 Table and include …*are from an Aboriginal or Torres Strait Islander*, *Polynesian*, *Asian or Middle Eastern background*. In fact, all seven instances group Aboriginal and Torres Strait Islander peoples with other identity categories:

- Chinese, Indian and the Pacific islands (2x),
- Mediterranean, Asian, Middle Eastern, Pacific Island,
- Vietnamese, Chinese, Middle Eastern, Polynesian and Melanesian,
- Asian, Middle Eastern, Polynesian, Mediterranean,
- Polynesian, Asian or Middle Eastern,
- Pacific island, Indian subcontinent or Chinese cultural background.

These associations are mostly in reference to groupings deriving from diabetes research or health programs, which journalists draw on in their writing. However, it could be argued that these linguistic practices of association have the potential to contribute to ‘the depiction of Indigenous people as “other”‘ [22, p. 92].

Second, the expression *… of Aboriginal appearance* implies that people are easily identifiable as Aboriginal by their skin colour and other aspects of appearance (facial features, hair, etc). It is again useful to quote the reporting guidelines in this respect:

Aboriginal and Torres Strait Islander peoples are diverse. They are not one homogenous group that think, speak and look the same. […] Like many cultural groups, Aboriginal and Torres Strait Islander peoples have diverse skin tones, features and appearances. It’s easy to make assumptions on whether you think someone ‘looks’ Aboriginal or Torres Strait Islander. […] As Gamilaroi woman and sociologist Dr Bindi Bennett puts it, “According to current media constructions, a ‘real’ Aboriginal person is dark-skinned, lives in a remote area of Australia and is in abject poverty.” Aboriginality is determined by a number of factors—cultural heritage, community recognition and descent. Skin tone does not reflect whether someone is recognised as an Aboriginal or Torres Strait Islander person. [1, p. 7]

In order to find out if the link between appearance and ethnicity/race is unique to the DNC and unique to Aboriginality, it is useful to briefly consult the second corpus: In NOW-OZ, the phrase ‘of [ETHNIC LABEL] appearance’ does occur with various labels, although it is infrequent (0.7 per million words). There are 250 instances of this expression in NOW-OZ, with different adjectives provided in the relevant slot before *appearance* (*Aboriginal*, *African*, *Asian*, *Asiatic*, *Caucasian*, *Chinese*, *European*, *Hispanic*, *Indian*, *Indian/Pakistani*, *Indian/Sub-continental*, *Jewish*, *Lebanese*, *Melanesian*, *Middle-Eastern*, *Muslim*, *south-asian*, *subcontinental*, *Sudanese*, *Uighur*). The vast majority (91.2%) of these expressions refer either to missing persons (56) or crime (172, including descriptions of victims or their bodies, perpetrators, and wanted persons/suspects). Thus, the expression ‘of [ETHNIC LABEL] appearance’ seems to be associated with police reports, in relation to crime or missing people. It is not surprising, then, that *of Aboriginal appearance* in the DNC is also associated with a missing person’s report:

[…]

Detective Acting Senior Sergeant Ian Kennon said Mr Cooper was reported missing about 8.30am on Thursday. […] “Mr Cooper is believed to be diabetic, however information suggests he was not in possession of any medication.” Mr Cooper is a 46-year-old man **of Aboriginal appearance**, about 180cm tall and of medium build with curly black and grey hair and a goatee. He was last seen wearing a pair of blue denim shorts with no shirt and was possibly carrying a yellow Dolphin brand torch. Anyone with information should call police on 131 444.(T15N001)

In other words, such problematic expressions seem to originate in police discourse, but are *reproduced* by the media.

A third expression worth briefly commenting on is that of *Aboriginal Australia*. In the DNC, it seems to be used as metonymic expression for Aboriginal and Torres Strait Islander peoples in Australia or in a particular region of Australia:

- Professor Alex Brown, head of the Aboriginal Research Unit at the South Australian Health and Medical Research Institute, said a majority of adults over 50 in some indigenous communities will have it. “It is probably the leading cause of preventable blindness **in Aboriginal Australia**,” he said. (V13N023)
- Life expectancy **in Aboriginal Australia** is on par with countries such as Azerbaijan, Vanuatu and Guatemala. (S13N011)
- The Alice Springs-based enterprise will aim to tackle a cancer-causing virus endemic **in indigenous central Australia**, its only significant instance outside South America and central Africa. (A17N005)

Van Leeuwen talks about such cases as *spatialization*, ‘a form of objectivation in which social actors are represented by means of reference to a place with which they are, in the given context, closely associated’ [31, p. 46]. He argues that objectivation is a type of impersonalization which can ‘background the identity and/or role of social actors; it can lend impersonal authority or force to an action or quality of a social actor; and it can add positive or negative connotations to an action or utterance of a social actor.’ [31, p. 47]. In the three examples above, it appears that the people who are affected are indeed backgrounded–whether this is a practice that should be seen critically here is another matter, since the reported matters are all negative (preventable blindness; low life expectancy; a rare virus). Another question is whether the expression *Aboriginal Australia* when it refers to people (rather than the country itself) nevertheless indirectly implies that there is such a thing as a ‘non-Aboriginal Australia’. The representation of Country and its relation to land rights is beyond the scope of this article, however.

### Collectivisation and differentiation

How a particular social group is discussed can influence the provision and use of health services [32, p. 134]. I therefore now examine more closely references to groups of people, with a focus on instances where *Aboriginal*, *Aboriginal and/or Torres Strait Islander*, and *Indigenous* pre-modify the lemma COMMUNITY. Previous analysis of newspaper discourse has suggested that ‘it is those who are labelled as a *community* that are represented as being outside the “unmarked” norm or even outside *society*’ [36, p. 59]. Using the example of *Muslim community*, Baker et al [37, p. 123] point out that this term is ‘potentially collectivising and differentiating’, as it can be used to ‘(a) collectivise Muslims into a single group and (b) differentiate them from others.’ (Although they do not cite him here, van Leeuwen [31] proposes collectivization and differentiation as categories in his social actor network.) In this way, a particular group is constructed as homogenous and distinctly separate from other groups, especially when it is used with the definite article, as in (uncritical) references to *the Muslim community* [37, p. 132] or, in this case, *the Aboriginal community* or *the Indigenous community*. The issue of treating Indigenous people as one undifferentiated group is one that has been commented upon previously:

‘Aboriginal and Torres Strait Islander peoples are diverse, have disparate views, perspectives and stories. […] Aboriginal and Torres Strait Islander people are not one monolithic, homogenous group […]’ [1, p. 3]

‘Of course there is no “indigenous community”–we are many and our issues myriad and diverse. But we know that we also share our fate and our connection runs deep.’ (Stan Grant, a Wiradjuri man and highly experienced journalist [38, p. 214])

‘Indigenous people are not a homogeneous group and our views reflect this diversity.’ Celeste Liddle, Arrernte woman and social commentator (cited in [1, p. 8])

**Fig 5:**
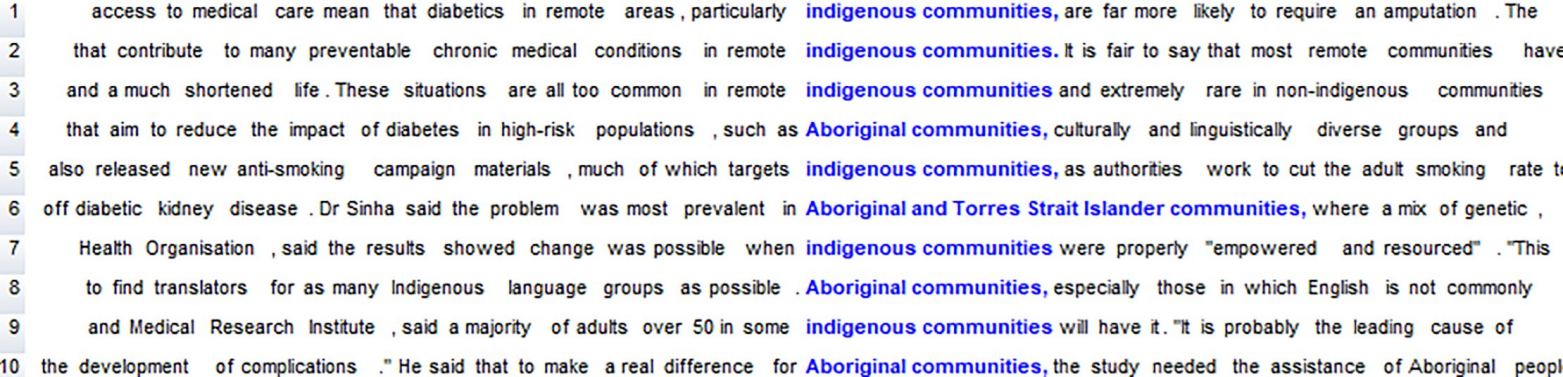
*Aboriginal/Aboriginal* and *Torres Strait Islander/Indigenous* communities.

All occurrences in the DNC of *Aboriginal/Aboriginal and/or Torres Strait Islander/Indigenous* + COMMUNITY are used uncritically (i.e. without scare quotes, adjectives such as *so-called*, etc). But, as Table 4 shows, about half of the 21 instances in 13 texts are in fact plural forms (10 occurrences). These ten plural occurrences do not presume that there is one homogenous Aboriginal or Indigenous community across Australia, but rather recognise the existence of different communities (Fig 5). (While the search terms do not capture instances with intervening words, there is only one such instance in the DNC (*Aboriginal people*, *communities and organisations*), which would bring the plural occurrences to 11. There is one instance of *non-Indigenous communities* in the DNC.)

**Table 4:** *Aboriginal/Aboriginal* and *Torres Strait Islander/Indigenous* + COMMUNITY.

Aboriginal and Torres Strait Islander communities	1
Aboriginal communities (3x), Aboriginal community (8x)	11
Indigenous communities (6x); Indigenous community (3x)	9
**Total (across 13 texts)**	**21**

However, there is little reference to any differences between communities, with only a few modifying the communities further (line 8: *Aboriginal communities*, *especially those in which English is not commonly spoken*). There are three co-textual references to remoteness (line 1: *diabetics in remote areas*, *particularly indigenous communities*; lines 2 & 3: *in remote indigenous communities*). While these distinguish remote ‘communities’ from others, they imply that all remote communities are homogenous. Note that the noun use *diabetics* in line 1 is in itself problematic [39].

Turning now to the eleven singular occurrences, one instance refers to an organisation’s name (*the National Aboriginal Community Controlled Health Organisation*). The term *Aboriginal Community Controlled Health Organisation* describes ‘organisations which provides holistic Primary Health Care and are run *by* Aboriginal and Torres Strait Islander people *for* Aboriginal and Torres Strait Islander people.’ [35, p. 1; italics in original]. There is also another use of *Aboriginal community-controlled* in reference to the health service sector, i.e. *a new central Australian academic health science centre*, *led by the Aboriginal community-controlled health service sector*. Here it is the health service sector that is defined through the definite article, rather than the Aboriginal community.

The majority of the remaining instances are references to Aboriginal people in a particular location (e.g. *in a central Australian indigenous community*; *after living in a remote Aboriginal community*). This is often only apparent when reading the extended co-text. Such location-specific uses are similar to the use of the plural, in that they recognise the existence of different groups within Australia.

Only three instances seem to imply the existence of a single homogenous Aboriginal or Indigenous community at the national level in Australia:

- THE Aboriginal community has one of the world’s highest rates of type 2 diabetes, up to four times the rate of other Australians. (V15N023)
- While poor diet and lack of exercise are typically blamed, growing evidence suggests the diabetes epidemic in the Aboriginal community has far more complex causes. (V15N021)
- … the decline had importance for the indigenous community (S13N011)

As a point of comparison, NOW-OZ contains 373 instances of *the Aboriginal community* in contrast to six instances of *the non-Aboriginal community*, and 358 instances of *the Indigenous community* compared to ten instances of *the non-Indigenous community*. The extent to which *the … community* occurs with other premodifiers in the relevant pre-noun slot (e.g. *Muslim*, *LGBTQI*, *CALD*, *type 1*) is worth comparing in future research.

In sum, highly collectivising/homogenising instances of *Aboriginal/Indigenous community* are rare in the DNC, perhaps because other expressions are also commonly used to refer to all Aboriginal and/or Torres Strait Islander people (e.g. … *people*, *Australians*, *population[s]*). In any case, people are clearly not represented as individuals with whom we might interact or identify with (individualization [31, p. 37], but rather as generic or specific groups of participants who may seem ‘symbolically removed from the readers’ world of immediate experience’ [31, p. 36]. Indeed, the majority of instances of *Aboriginal/Indigenous* are not used to refer to individuals but rather to groups. Only in some cases are these words co-textually associated with individuals (e.g. *Justin Mohamed*, *chair of the National Aboriginal Community Controlled Health Organisation; Ms Mundy … a boost for indigenous Australians like her*). Whether this phenomenon is unique to Aboriginal and/or Torres Strait Islander people or not is a matter for future research. It could well be the case that diabetes coverage tends to be de-individualised in general.

In relation to health news, it may sometimes appear necessary to group people together for the purpose of referring to statistics or other scientific findings (as I have also done in this article). In addition, it is important to note that expressions such as *Indigenous communities* or *the Indigenous community* are not just used by ‘outsiders’, but also by cited Aboriginal sources (if we assume the newspaper is accurately representing the sources’ words):

- Citing improvements in smoking levels, Justin Mohamed, chair of the National Aboriginal Community Controlled Health Organisation, said the results showed change was possible when **indigenous communities** were properly “empowered and resourced”. [Justin Mohamed is a Gooreng Gooreng man from Bundaberg in Queensland] (S13N011)
- Lisa Jackson Pulver, professor of public health and director of Muru Marri indigenous health unit at the University of NSW [sic], said the decline had importance for **the indigenous community** [Lisa Jackson Pulver is a Wiradjuri woman] (S13N011)

I suggest that collectivising references may have a role to play for an in-group, in particular in relation to the fight for rights and recognition. Aboriginal identity can unite and bring together people. But this is not how such references are typically used in the print media I surveyed here. In addition, this is most likely an area where people’s opinions on language use varies. The term *community* is interesting precisely because of its ambiguous evaluative potential, and it is important to consider whether it is used by insiders in self-descriptions or by outsiders for Othering [36].

### References to (groups of) people: Qualitative analysis

As mentioned above, an important sub-category of the use of *Aboriginal/Indigenous* consists of references to [groups of] people. Let us now look more closely at how these references are used, by summarising the results from the concordance analysis. More specifically, I examined the expressions in Table 5 qualitatively for the co-text in which they occur (79 total instances; see S1 Table). I will illustrate each identified category with examples from the DNC.

**Table 5:** References to [groups] of people.

‘people’	*Aboriginal and Torres Strait Islander people; Aboriginal people*, *Aboriginal or Torres Strait Islander people*, *Indigenous people; Indigenous peoples*	26
‘community’	*Aboriginal and Torres Strait Islander communities*, *Aboriginal communities*, *Indigenous communities*, *Indigenous community Aboriginal community*	21
‘background’	*BE from … background* [see S2 Table for variants]	7
‘population’	*Indigenous population; Indigenous populations*	6
‘Australian’	*Aboriginal Australian; Indigenous Australians*	9
‘being’	*being Aboriginal or Torres Strait Islander*, *… was Aboriginal*, *are indigenous*	4
Other	*an indigenous child*, *Aboriginal women*, *Indigenous patients*, *Indigenous groups*, *Indigenous lives*, *Indigenous* [noun, i.e. *between indigenous and non-indigenous*]	6
**Total**		**79**

The vast majority of the 79 instances refer to Aboriginal and Torres Strait Islander people in relation to a higher risk, likelihood, or incidence of having or developing diabetes (or complications/effects). The use of such statistics is regarded in [22] as contributing to the dominant theme of failure in Australian representations of Aboriginal and Torres Strait Islander health. In relation to **risk**, different phraseologies can be identified in the corpus:

*Being at risk/there being risk*:

- You are more **at risk** if you: […] Are from an Aboriginal or Torres Strait Islander, Polynesian, Asian or Middle Eastern background (W15N025)
- Like other indigenous groups, our indigenous people are **at** very high **risk** of developing diabetes, with rates between three and four times higher than in non-indigenous people. (H13NN010)
- People living in Blacktown are three times as likely to develop diabetes as those living in Mosman. Part of this may be due to the region having higher proportions of Indigenous people, people with mental health problems and people in their childbearing years, who are **at** higher **risk**. (N16N006)
- According to Diabetes Australia, which has a risk calculator on its website, **there is** increased **risk** if you: […] are from an Aboriginal or Torres Strait Islander background; […]. (A17N003)
- INDIGENOUS people who are **at risk** of becoming blind because of diabetes are receiving world-class healthcare for the first time. (A13N004)

Having a higher risk of

- Women from some ethnic backgrounds have a higher **risk** of developing gestational diabetes, these include: Aboriginal and Torres Strait Islander, Vietnamese, Chinese, Middle Eastern, Polynesian and Melanesian women. (W16N004)

Ethnic identity/background being a risk factor

- Family history, age and being Aboriginal or Torres Strait Islander are also **risk factors**. (R15N004)
- While there are a number of **risk factors** associated with developing gestational diabetes, including being over 30 years of age, being overweight, having a family history of type 2 diabetes and being from a particular ethnic background (including Asian, Middle Eastern, Polynesian, Mediterranean, Aboriginal and Torres Strait Islander) […]. (V16NN001)

*High-risk genetic backgrounds or population*:

- […] people from **high-risk** genetic backgrounds, such as indigenous, Chinese, Indian and the Pacific islands. […]” (G13NN002; C13NN009)
- […] the impact of diabetes in **high-risk** populations, such as Aboriginal communities, culturally and linguistically diverse groups and people living in rural and remote areas of WA. (W15NN010)

References to **likelihood** tend to be comparative and use three similar constructions:

- BE (much/far) more likely to get/have (type) diabetes [or diabetes-related effects]
- BE X times more likely as/than [comparison group] to have diabetes [or diabetes-related effects]
- BE X times more likely to get/have diabetes [or diabetes-related effects] than/compared with [comparison group]

While these refer to the same statistics as the ‘risk’ occurrences, lexicalisation with ‘likelihood’ seems preferable. Clearly, it is highly problematic to construct being an Aboriginal and Torres Strait Islander person as ‘high-risk’. One of the items on the ‘Blackfulla Test’ for Indigenous health research grants/publications states that a paper/proposal fails if it ‘**[r]efers to Indigenous peoples primarily in terms of “risk” and “vulnerability”** or worse describes Indigeneity as the risk factor’ [40, bold in original]. It must be noted that there is likely a general association between the phrase at *** risk (from/of) and *diabetes* in newspaper coverage, and that much *at risk*-language occurs in the context of health and illness more generally, potentially inherited from health research [41].

There are also many instances that refer to Aboriginal and Torres Strait Islander people as having high **incidence** of diabetes, complications of diabetes, dying from diabetes, etc. These usually contain numbers (e.g. ***One in six*** *Aboriginal or Torres Strait Islander people aged 25 and over have diabetes or high sugar levels*; *It* [having elevated blood sugar levels] *could be as high as* ***30 per cent*** *among indigenous Australians*), and often include a comparison group (e.g. *Among Aboriginal and Torres Strait Islander people*, *Type 2 diabetes is seen at rates three to four times higher* ***than in other Australians***; *The death rate from diabetes among indigenous people is almost 12 times* ***that of non-indigenous people***). Such uses could again be seen as differentiating.

In addition to risk, likelihood and incidence of diabetes-related health issues, there are several references to other health issues that affect Aboriginal or Torres Strait Islander people in negative ways, for example in relation to life expectancy, heart valves, kidney disease, smoking rates, preventable chronic medical conditions, HTLV-1 infection. References to the gap in life expectancy are aligned with the prominent *Indigenous health crisis* frame and also found in Australian news from the 1990s [16, p. 27].

In some cases, reference is made to the difficulties that are encountered by Aboriginal and Torres Strait Islander people, e.g.:

- “Aboriginal people don’t want to see a GP unless they really have to. You (city people) do take it for granted that you can get to see a doctor,” Ms Mundy says. “A lot of them don’t bulk bill as well so it’s **hard** for us as Aboriginal people and other people with not a lot of money (to see them).” (A13N002)
- Wischer became interested in an online business model after living in a remote Aboriginal community and gaining weight following the birth of her children. She experienced first hand the **difficulty** of both accessing healthy food and exercising in extreme temperatures in a remote area. (H14N019)

Such references can be classified under negativity in that they refer to difficulties and problems. It must be pointed out that the second example here refers to remoteness as a major social determinant of health, which can affect individuals regardless of their ethnicity.

In sum, the vast majority (49 instances) of the 79 instances occur in contexts to do with negativity, with only 24 occurring in positive, neutral, or ambiguous contexts (S1 Table). There is no doubt that the use of *Aboriginal/Indigenous* for references to [groups of] people has a negative semantic prosody. More generally, the attention paid to rates of health conditions or diseases in Indigenous peoples is part of a media theme or frame, which Tong et al [14, p. 163] call ‘threatening the vulnerable’ (where the health condition is framed as affecting vulnerable groups). They found that the news media compare high rates of chronic kidney disease among Indigenous peoples with ‘the general population’ [14, p. 163]. It is possible that such reporting can encourage individuals to seek advice. However, regarding the mainstream news media it is ‘difficult to assess the extent of its relevance and effectiveness in minority communities’ [14, p. 167], and this would have to be the subject of additional study, incorporating a wider range of media types, including Indigenous media.

There are only a few exceptions where instances are neutral or positive. Occurrences classified as neutral often have to do with recruitment or participation in studies (e.g.: *she wanted to engage the Aboriginal community to take part in more trials*; *Researchers hope to recruit 4000 Aboriginal people across South Australia*; *The program will target … Aboriginal and Torres Strait Islander people aged 18 and over*.). Another small group refers to improvements in health care or technology, or making help a priority e.g.:

- Ms Mundy says the trial shows that blood sugar monitoring can be done successfully from the comfort of one’s home—**a boost for indigenous Australians like her**. (A13N002)
- Fred Hollows Foundation chief executive Brian Doolan said the technology was **the next big jump in the delivery of health services to indigenous Australians since Hollows’s time**. “It puts the best of technology right into the hands of Aboriginal healthcare workers, the people right on the frontline,” he said. (A13N004)
- So **the announcement of a new central Australian academic health science centre, led by the Aboriginal community-controlled health service sector** and bringing together a consortium of 11 clinical and research groups, is a big deal for her and many women like her. (A17N005)
- “**Helping** people living in rural and remote locations, **Aboriginal and Torres Strait Islander people**, and people from culturally and linguistically diverse backgrounds **will also be a priority**,” Mr Dick said. [R16N007]

However, since the positivity is primarily associated with initiatives by governments and charities (also found in research on other health issues [9, 18, 22]), such positive stories are arguably not empowering and do not contribute to shifting the focus from deficit to a strength-based approach, which ‘recognises the resilience of individuals and focuses on their potential, strengths, interests, abilities, knowledge and capacities’ [42, p. 649].

While not in relation to diabetes, but rather with respect to smoking levels, two quotations do directly reference the possibility for positive change, and are made by the two experts already mentioned above:

- Lisa Jackson Pulver, professor of public health and director of Muru Marri indigenous health unit at the University of NSW, said the decline [in smoking] had importance for the indigenous community because those who had quit or never smoked would have probably grown up in households with much higher rates of smoking. “These are people whose odds, according to all the reports and everything we know, were that they were going to smoke too… Now the hard-core stats are showing that doesn’t have to be you,” she said. “This is something very important for Aboriginal people, who so often get told because you’re in that environment, this is what’s going to happen.” (S13N011)
- Citing improvements in smoking levels, Justin Mohamed, chair of the National Aboriginal Community Controlled Health Organisation, said the results showed change was possible when indigenous communities were properly “empowered and resourced”. “This report should make government realise that things can change… This shows that when we’re given control, we can win,” he said. (S13N011)

This illustrates how the media theme of ‘fatalism’ [22, p. 92] can be countered and shows the importance of not just referring to Aboriginal and Torres Strait Islander people(s) and issues, but of incorporating their voices. This also includes publications, meaning that the approach taken in this article needs to be complemented with studies that examine how Aboriginal and Torres Strait Islander peoples *themselves* experience, conceptualise, and talk about diabetes (see [43] on such an approach in relation to dementia caregiving).

## Conclusion

The use of language in health is regarded as highly significant, as it constructs and reproduces problems and solutions, value systems and beliefs, blame and responsibility (e.g. [32, p. 125–126]. This article has shown that Aboriginal and Torres Strait Islander people(s) and issues lack in visibility in diabetes coverage, and continue to be associated with deficit framing. This result is in line with research on coverage of other health issues, showing the Australian media’s perpetuation of a ‘discourse of deficits’ [18, p. 282]. Where *Aboriginal* or *Indigenous* are used to refer to [groups of] people, such mentions occur in the context of negativity, often in relation to risk. A media focus on negative aspects of Indigenous health may lead to awareness, research, and programs, but can also reinforce stereotypes and stigma [23]. Such emphasis on negativity can be disempowering:

Many years and many millions of research dollars have been invested in defining, quantifying and cataloguing the negatives—disgraceful health outcomes, the inability of many existing programs to achieve any real improvements, and the sequelae of continued disadvantage and intergenerational grief. Too many of our young people are caught up in emotional turmoil, reinforced by societal structures that inhibit their aspirations. [44]

This negativity also has the potential to reinforce discourses that represent the health disadvantage experienced by Aboriginal and Torres Strait Islander people(s) as a ‘problem’ (Aldrich et al identify this discourse in Australian politicians’ statements in [32]). There have been many calls to focus on success rather than disadvantage (e.g. by Stan Grant and Tom Calma, cited in [18]) and ‘to remove the deficit lens’:

We are asking to be seen as the dynamic, resilient and self-determining people that we are. We know that there is a lot of work to do, but today we want to celebrate success and highlight our strengths and achievements. We want to acknowledge the incredible work being carried out in Aboriginal and Torres Strait Islander communities across the country. The 2019 Close the Gap report–Our Choices, Our Voices, prepared by the Lowitja Institute, highlights a number of organisations improving the health and wellbeing of our peoples. They show that when Aboriginal and Torres Strait Islander people are involved in the design and delivery of the services they need, we are far more likely to achieve success. [45]

The guidelines therefore ask newsrooms to aim for ‘a balance between “bad news” and “good news”‘ [1, p. 14]. Similarly, guidelines from the Public Health Association Australia state:

It is important that when writing about Aboriginal and Torres Strait Islander people that the tone and framing is not deficit focused. Many Aboriginal and Torres Strait Islander people face disadvantage however there are many strengths within communities. It is important to reflect those strengths in the language we use. [35, p. 1]

Both Negativity (bad news) and Positivity (good news) are news values that can be established through language to construct newsworthiness [30]. While Negativity has been called ‘the basic news value’ [46, p. 156], there is clearly room in health news for Positivity, e.g. in stories about medical breakthroughs [14], successful programs [9] or stories that foreground the positive role of Aboriginal health workers [16]. However, the positivity should be in line with a strength-based approach, recognising the capacities, knowledge, and resilience of Aboriginal and Torres Strait Islander people [24, 42]. Thus, to change the discourse would require both an increased visibility as well as a change in the deficit frame. To bring about such change, the various causes and practices that can explain this invisibility and focus on negativity should be the subject of research, not limited to journalism but also considering health research, health policy, and health funding. Pyett et al [23], for instance, make a number of suggestions for decolonising health promotion and research, while others have argued that health research should examine improvements, not just deficits, and challenge disempowering myths of failure [42, 47]. McCallum [17] has examined the complex relationships between news media coverage and Indigenous policy development, while Sweet et al [24] propose that clear benefits may come from adapting decolonising practices from the health sector for journalism education and practice. Different initiatives should also be supported and evaluated for their impact–for example, the Aboriginal and Torres Strait Islander newspaper *The Koori Mail* has represented health issues successfully [9], and the *Guardian* has partnered with *Indigenous X* to feature diverse Indigenous voices and opinions.

There are several limitations to this study. It did not systematically analyse frames or undertake full-text analysis, and it focused only on newspapers rather than other media. Despite starting with almost 700 newspaper items, the low presence of mentions of Aboriginal and Torres Strait Islander people(s) and issues resulted in a small dataset, which brings with it inherent limitations. Further, the study did not analyse whether Aboriginal and Torres Strait Islander people are represented *more* negatively than other social groups. Given the general importance of the news value of Negativity, we would expect negative reporting in relation to a wide range of topics and groups. In the analysis of *association* for example, we have seen that other groups (e.g. Middle Eastern, Polynesian, Chinese) may occur in similar co-texts. While the lack of comparison is a limitation of this study, it is worth pointing out that, from a historical perspective, no other social group in Australia has the same past and ongoing experiences of colonisation as Aboriginal and Torres Strait Islander people. To give just two examples that are well-known in Australia but perhaps less known elsewhere: Until 1970 many Indigenous children were taken by force from their parents (the ‘Stolen Generations’), and the 2007 Northern Territory Emergency Response (the ‘Intervention’) involved the use of the military to enforce various coercive measures in remote NT communities.

## Supporting information

S1 Table Concordance-based analysis of semantic prosody. (DOCX)

S2 Table Uses of *Aboriginal* and *Indigenous* in the DNC. (DOCX)
