# Supplementary material for: Invisible or high-risk: Computer-assisted discourse analysis of references to Aboriginal and Torres Strait Islander people(s) and issues in a newspaper corpus about diabetes
Source: PLoS One. 2020 Jun 11;15(6):e0234486. doi: 10.1371/journal.pone.0234486 (PMC7289392; doi:10.1371/journal.pone.0234486)
Supplement: S1 Table — (DOCX) [file pone.0234486.s001.docx]

**S1 Table. Concordance-based analysis of semantic prosody.**

| **Instances identified as having a negative co-text (n = 49)** | |
| --- | --- |
| 1. You are more at risk if you: […] Are from **an Aboriginal or Torres Strait Islander**, Polynesian, Asian or Middle Eastern **background** 2. Like other indigenous groups, **our indigenous people** are at very high risk of developing diabetes, with rates between three and four times higher than in non-indigenous people. 3. People living in Blacktown are three times as likely to develop diabetes as those living in Mosman. Part of this may be due to the region having higher proportions of **Indigenous people**, people with mental health problems and people in their childbearing years, who are at higher risk. 4. According to Diabetes Australia, which has a risk calculator on its website, there is increased risk if you: […] are from **an Aboriginal or Torres Strait Islander background**; […]. 5. Women from some ethnic backgrounds have a higher risk of developing gestational diabetes, these include: **Aboriginal and Torres Strait Islander**, Vietnamese, Chinese, Middle Eastern, Polynesian and Melanesian women. 6. Family history, age and being **Aboriginal or Torres Strait Islander** are also risk factors. 7. While there are a number of risk factors associated with developing gestational diabetes, including being over 30 years of age, being overweight, having a family history of type 2 diabetes and being from a particular ethnic background (including Asian, Middle Eastern, Polynesian, Mediterranean, **Aboriginal and Torres Strait Islander**)… 8. […] people from high-risk genetic backgrounds, such as **indigenous**, Chinese, Indian and the Pacific islands. […]” 9. […] people from high-risk genetic backgrounds, such as **indigenous**, Chinese, Indian and the Pacific islands. [repeated across newspapers] 10. […] the impact of diabetes in high-risk populations, such as **Aboriginal communities**, culturally and linguistically diverse groups and people living in rural and remote areas of WA. 11. In WA, **Aboriginal people** were 38 times more likely to undergo a major leg amputation [a complication of diabetes] compared with non-indigenous people. 12. **Indigenous people** are about three times more likely to have diabetes, 10 times more likely to be admitted for diabetic foot complications and 30 times more likely to suffer diabetes-related lower limb amputation than non-indigenous people. 13. According to an ABC Radio report, **Aboriginal and Torres Strait Islander people** are three times more likely as non-indigenous people to have diabetes. 14. Regrettably, i**ndigenous Australians** are 3.5 times more likely than other Australians to have diabetes, and four times more likely to be hospitalised or die from the condition. 15. … and **indigenous Australians** are more than three times as likely as non-indigenous Australians to have diabetes. 16. I**ndigenous Australians** were the most vulnerable. “They are three times more likely to get diabetes than a Caucasian person,” he said. 17. Risk factors and issues surrounding access to medical care mean that diabetics in remote areas, particularly **indigenous communities**, are far more likely to require an amputation [a complication of diabetes]. 18. … with the **Australian indigenous population** between two times and six times more likely to have diabetes 19. **We are indigenous** so more likely to get it [type 2 diabetes], but I didn’t expect it this young 20. **We are indigenous** so more likely to get it [type 2 diabetes], but I didn’t expect it this young [repeated across newspapers] | High(er) risk or likelihood (diabetes-related) |
| 1. One in six **Aboriginal or Torres Strait Islander people** aged 25 and over have diabetes or high sugar levels 2. It [having elevated blood sugar levels] could be as high as 30 per cent among **indigenous Australians** 3. Among **Aboriginal and Torres Strait Islander people**, Type 2 diabetes is seen at rates three to four times higher than in other Australians; 4. The death rate from diabetes among **indigenou**s people is almost 12 times that of non-indigenous people 5. The rate of diabetes in **Aboriginal people** was three times as high than in non-Indigenous people. 6. Alice Springs mother Nellie Impu is part of a grim health statistic profoundly out of place in a first-world nation: one in five pregnant **Aboriginal women** in the Northern Territory has diabetes. 7. Dr Sinha said the problem was most prevalent in **Aboriginal and Torres Strait Islander communities**, where a mix of genetic, lifestyle and socio-economic factors were involved. 8. Type 2 diabetes usually develops over the age of 45 but is becoming increasingly common in younger age groups and **Aboriginal and Torres Strait Islander people**. 9. “With **Aboriginal and Torres Strait Islander people**, we’re seeing high rates of diabetes, high levels of smoking, and high levels of overweight and obesity, which are all risk factors for heart disease,” 10. Dr Barclay said diabetes was common in people from Mediterranean, Asian, Middle Eastern, Pacific Island and **indigenous Australian** **backgrounds** 11. These situations [diabetes, kidney complications] are all too common in remote **indigenous communities** and extremely rare in non-indigenous communities. 12. **Aboriginal communities**, especially those in which English is not commonly spoken, are hit disproportionately hard by type 2 diabetes. 13. It is critical that governments are aware of the need for action before a large percentage of the **indigenous population** is lost to largely preventable complications [from diabetes]. 14. Professor Alex Brown, head of the Aboriginal Research Unit at the South Australian Health and Medical Research Institute, said a majority of adults over 50 in some **indigenous communities** will have it [diabetes]. 15. While poor diet and lack of exercise are typically blamed, growing evidence suggests the diabetes epidemic in the **Aboriginal community** has far more complex causes. 16. THE **Aboriginal community** has one of the world’s highest rates of type 2 diabetes, up to four times the rate of other Australians. 17. “Diabetes particularly type 2 is occurring much earlier in young i**ndigenous people** in the NT,” she said. 18. Complications of diabetes include heart, eye, foot and kidney disease, and complication rates in the **indigenous population** are among the highest in the world and about 10 times non-indigenous rates. | High(er) incidence (diabetes related) |
| 1. An **indigenous child** born today can expect to live 71.4 years, far lower than the expected national average of 82.1 years. 2. 71.4 Life expectancy of an **Aboriginal Australian** born today 82.1 Life expectancy for a non-Aboriginal Australian born today [comparison of life expectancy] 3. **Indigenous Australians** are nearly four times as likely to have kidney disease and three times as likely to have experienced high or very high levels of psychological distress. 4. The program will also address the soaring demand for dialysis in remote communities, with **indigenous Australians** five times as likely to have end-stage kidney disease than other Australians. 5. A recent study found HTLV-1 infection rates in a central A**ustralian indigenous community** of more than 40 per cent. 6. … but more than 40 per cent of i**ndigenous people** aged 15 and over are smoking on a daily basis, compared with less than 19 per cent of non-indigenous people. 7. It [rheumatic fever] was also still a big concern in **indigenous people**. 8. “(Heart valves) become thick and narrowed and leak in later life so by the time people are 40, 50, 60 they have a real problem with their heart valves. In the **indigenous population** this can happen earlier - in their 20 and 30s,” he said. | Negatively affected (other health issues) |
| 1. “A lot of them don’t bulk bill as well so it’s hard for us as **Aboriginal people** and other people with not a lot of money (to see them). 2. Wischer became interested in an online business model after living in a remote **Aboriginal community** and gaining weight following the birth of her children. She experienced first hand the **difficulty** of both accessing healthy food and exercising in extreme temperatures in a remote area. 3. For most Australians, it would be difficult to imagine the general health circumstances, lack of facilities and resources that contribute to many preventable chronic medical conditions in remote **indigenous communities**. | Difficulties |
| **Instances identified as having a positive, ambiguous, or neutral co-text (n = 24)** | |
| 1. Ms Mundy says the trial shows that blood sugar monitoring can be done successfully from the comfort of one’s home - a boost for **indigenous Australians** like her. [positive] 2. Citing improvements in smoking levels, Justin Mohamed, chair of the National Aboriginal Community Controlled Health Organisation, said the results showed change was possible when **indigenous communities** were properly “empowered and resourced”. [positive] 3. Lisa Jackson Pulver, professor of public health and director of Muru Marri indigenous health unit at the University of NSW, said the decline had importance for the **indigenous community** because those who had quit or never smoked would have probably grown up in households with much higher rates of smoking. [positive] 4. Strands of NBN improving **indigenous lives** [positive] 5. Fred Hollows Foundation chief executive Brian Doolan said the technology was the next big jump in the delivery of health services to **indigenous Australians** since Hollows’s time [positive or neutral] 6. “This [decline in smoking] is something very important for **Aboriginal people**, who so often get told because you’re in that environment, this is what’s going to happen.” [positive or neutral] 7. Researchers hope to recruit 4000 **Aboriginal people** across South Australia to take part - 2000 with diabetes and 2000 without - to better understand its causes and complications. [neutral, though does imply that they are affected] 8. He said that to make a real difference for **Aboriginal communities**, the study needed the assistance of Aboriginal people, communities and organisations across SA. [neutral, two different instances in the same concordance line] 9. He said that to make a real difference for Aboriginal communities, the study needed the assistance of **Aboriginal people**, communities and organisations across SA. [neutral, two different instances in the same concordance line] 10. “We’re really trying to work with our **Aboriginal community** now for the best way to help feel comfortable about it because our project team needs to understand the experience of cancer for Aboriginal people” [neutral, two different instances in the same concordance line] 11. “We’re really trying to work with our Aboriginal community now for the best way to help feel comfortable about it because our project team needs to understand the experience of cancer for **Aboriginal people**” [neutral, two different instances in the same concordance line] 12. Dr Wales said one in five people in Armidale **was Aboriginal**, and she wanted to engage the Aboriginal community to take part in more trials, such as a new cancer-related telehealth pilot. [neutral] 13. The program will target people aged 45 and over, **Aboriginal and Torres Strait Islander people** aged 18 and over, and people living with a pre-existing condition that puts them at a high risk of developing a chronic disease. [neutral] 14. “Ms Ley also released new anti-smoking campaign materials, much of which targets **indigenous communities**, as authorities work to cut the adult smoking rate to 10 per cent by 2018. [neutral] 15. Armidale Community Health’s Coralie Wales, who is the project leader, is pleased with the results of the trial, which she said had a large number of participants from the **indigenous community**. [neutral] 16. “We want people to stay healthy and if they do have symptoms to get really early help,” Dr Wales said. “That’s what we’re working with the **Aboriginal community** to achieve.” [neutral] 17. … and she wanted to engage the **Aboriginal community** to take part in more trials, such as a new cancer-related telehealth pilot. [neutral] 18. An initiative funded by the Fred Hollows Foundation and rolled out this week now means that when **indigenous patients** - often from remote communities - come in to a health clinic, a retinal camera will send images for examination to a centre in Melbourne. [neutral] 19. … it was hoped to recruit one in six **Aboriginal people** over the age of 15 in SA to help with the study. [neutral] 20. … the study hoped to recruit one in six **Aboriginal people** over the age of 15 in SA [neutral] 21. “Helping people living in rural and remote locations, **Aboriginal and Torres Strait Islander people,** and people from culturally and linguistically diverse backgrounds will also be a priority,” Mr Dick said. [ambiguous, since help implies vulnerability] 22. It follows research released last week that showed the gap in life expectancy between **indigenous** and non-indigenous had declined slightly, to about 11 years. [ambiguous, includes negative (the gap) and positive (decline)] 23. **INDIGENOUS people** who are at risk of becoming blind because of diabetes are receiving world-class healthcare for the first time. [ambiguous, includes negative (risk of blindness) and positive (world-class healthcare)] 24. “**Aboriginal people** don’t want to see a GP unless they really have to. …” [ambiguous, could be read as negative by some readers] | |
| **Other (irrelevant or unrelated instances) (n = 6)** | |
| 1. Like **other indigenous groups**, our indigenous people are at very high risk of developing diabetes, with rates between three and four times higher than in non-indigenous people. [this instance does not refer to the Australian context, only the second noun group does, which is already included above] 2. **Indigenous populations** around the globe have a three to six times higher risk of type 2 diabetes. [not just in Australia] 3. Citing improvements in smoking levels, Justin Mohamed, chair of the **National Aboriginal Community Controlled Health Organisation**, [not a reference to community] 4. So the announcement of a new central Australian academic health science centre, led by the **Aboriginal community-controlled health service sector** [not a reference to community] 5. It [the atlas] also shows the heavy impact the disease is having on **indigenous peoples** around the world as lifestyles and diet change. [not just in Australia] 6. Genetics is a key driver of the disease. **Indigenous populations** around the world are at much higher risk of diabetes…. [not just in Australia] | |
