# Supplementary material for: Invisible or high-risk: Computer-assisted discourse analysis of references to Aboriginal and Torres Strait Islander people(s) and issues in a newspaper corpus about diabetes
Source: PLoS One. 2020 Jun 11;15(6):e0234486. doi: 10.1371/journal.pone.0234486 (PMC7289392; doi:10.1371/journal.pone.0234486)
Supplement: S2 Table — (DOCX) [file pone.0234486.s002.docx]

**S2 Table. Uses of *Aboriginal* and *Indigenous* in the DNC.**

| **Uses** | **Freq.** |
| --- | --- |
| Aboriginal people (10); Indigenous people (8x); Indigenous peoples (1x); Aboriginal and Torres Strait Islander people (6x); Aboriginal or Torres Strait Islander people (1x) | 26 |
| Aboriginal communities (3x); Aboriginal community (8); Indigenous communities (6x); Indigenous community (3x); Aboriginal and Torres Strait Islander communities (1x) | 21 |
| Indigenous Australians (8x); Aboriginal Australian (1x) | 9 |
| BE from … background (*are from an* ***Aboriginal or Torres Strait Islander background***; *being from a particular ethnic* ***background*** *(including Asian, Middle Eastern, Polynesian, Mediterranean,* ***Aboriginal and Torres Strait Islander****)*; *are from an* ***Aboriginal or Torres Strait Islander****, Polynesian, Asian or Middle Eastern* ***background***; *Women from some ethnic* ***backgrounds*** *have a higher risk of developing gestational diabetes, these include:* ***Aboriginal and Torres Strait Islander****, Vietnamese, Chinese, Middle Eastern, Polynesian and Melanesian women;* people from Mediterranean, Asian, Middle Eastern, Pacific Island and **indigenous Australian** **backgrounds**; people from high-risk genetic **backgrounds**, such as **indigenous**, Chinese, Indian and the Pacific islands 2x duplicated) | 7 |
| Indigenous population (4 x); Indigenous populations (2x) | 6 |
| BE … [we are indigenous 2x duplicated; being Aboriginal or Torres Strait Islander, was Aboriginal] | 4 |
| … health [Indigenous health (2x); Aboriginal and Torres Strait Islander health 1x] | 3 |
| … Australia [Aboriginal Australia 2x; Indigenous central Australia] | 3 |
| Aboriginal Health Theme Leader | 2 |
| The Aboriginal Research Unit; Wardliparingga Aboriginal Research Unit | 2 |
| Indigenous diets (2x duplicated) | 2 |
| … of Aboriginal appearance | 1 |
| Aboriginal Diabetes Study | 1 |
| Aboriginal healthcare workers | 1 |
| an Aboriginal liaison officer | 1 |
| the Aboriginal Medical Services Alliance Northern Territory | 1 |
| Aboriginal women | 1 |
| Aboriginal rock art | 1 |
| federal Indigenous Health Minister | 1 |
| director of Muru Marri indigenous health unit | 1 |
| Indigenous health issues | 1 |
| Indigenous health care | 1 |
| Indigenous diabetes | 1 |
| Indigenous smoking rates | 1 |
| Indigenous measures | 1 |
| Indigenous lives | 1 |
| Indigenous patients | 1 |
| Indigenous groups | 1 |
| an indigenous child | 1 |
| Indigenous [noun, i.e. the gap in life expectancy between indigenous and non-indigenous] | 1 |
| Indigenous language groups | 1 |
